# Transcriptome reprogramming in the shoot apical meristem of CymRSV‐infected *Nicotiana benthamiana* plants associates with viral exclusion and the lack of recovery

**DOI:** 10.1111/mpp.12875

**Published:** 2019-09-27

**Authors:** Anna Medzihradszky, Péter Gyula, Anita Sós‐Hegedűs, György Szittya, József Burgyán

**Affiliations:** ^1^ Department of Plant Biotechnology National Agricultural Research and Innovation Centre Szent‐Györgyi Albert u. 4 Gödöllő 2100 Hungary

**Keywords:** CymRSV, *Nicotiana benthamiana*, shoot apical meristem, silencing, transcriptome

## Abstract

In some plant–virus interactions plants show a sign of healing from virus infection, a phenomenon called symptom recovery. It is assumed that the meristem exclusion of the virus is essential to this process. The discovery of RNA silencing provided a possible mechanism to explain meristem exclusion and recovery. Here we show evidence that silencing is not the reason for meristem exclusion in *Nicotiana benthamiana* plants infected with Cymbidium ringspot virus (CymRSV). Transcriptome analysis followed by *in situ* hybridization shed light on the changes in gene expression in the shoot apical meristem (SAM) on virus infection. We observed the down‐regulation of meristem‐specific genes, including *WUSCHEL* (*WUS*). However, *WUS* was not down‐regulated in the SAM of plants infected with meristem‐invading viruses such as turnip vein‐clearing virus (TVCV) and cucumber mosaic virus (CMV). Moreover, there is no connection between loss of meristem function and fast shoot necrosis since TVCV necrotized the shoot while CMV did not. Our findings suggest that the observed transcriptional changes on virus infection in the shoot are key factors in tip necrosis and symptom recovery. We observed a lack of *GLYCERALDEHYDE 3-PHOSPHATE DEHYDROGENASE *(*GAPDH*) expression in tissues around the meristem, which likely stops virus replication and spread into the meristem.

The outcome of virus infections of plants may be symptom recovery, which is characterized by the emergence of new leaves showing attenuated or no symptoms. Symptom recovery is often associated with the exclusion of viruses from plant shoot apical meristems (SAM), but the underlying mechanisms of virus exclusion are still poorly understood. Recovery of plants from virus‐induced symptoms is one of the oldest mysteries in plant virology. The earliest report describing a plant defence mechanism was published almost 100 years ago, describing plant recovery from virus infection (Wingard, 1928). Infection of tobacco plants with tobacco ringspot nepovirus (ToRSV) induced very strong necrotic symptoms in the initially infected leaves, but later these plants recovered from the disease. The upper newly developed leaves looked healthy and were resistant to secondary infection of the same virus. At that time there was no explanation for this very interesting phenomenon. After the discovery of RNA silencing as a defence mechanism against invading molecular parasites, it was shown that RNA silencing plays a key role in the development of plant recovery from virus disease (Ghoshal and Sanfaçon, 2015; Ratcliff *et al.*, 1997; Silhavy *et al.*, 2002).

RNA silencing is a conserved eukaryotic pathway involved in almost all cellular processes, such as development, stress responses and genome defence. RNA silencing relies on the 21–24 nucleotide (nt) short interfering RNAs (siRNAs) and microRNAs (miRNAs), the hallmark molecules of silencing (Brodersen and Voinnet, 2006; Hamilton and Baulcombe, 1999; Llave, 2010; Parent *et al.*, 2012). These siRNAs have been shown to be mobile and to play an important role in the cell‐to‐cell and long‐distance (systemic) spreading of silencing (Dunoyer *et al.*, 2005; Havelda *et al.*, 2005; Schwach *et al.*, 2005). Viruses can suppress RNA silencing in different ways, from interfering with its initiation to causing an arrest in the assembly of a functional RNA‐induced silencing complex (RISC) (Burgyán and Havelda, 2011; Csorba *et al.*, 2015). One of the best characterized viral‐encoded silencing suppressor proteins (VSR) is the p19 protein of Cymbidium ringspot virus (CymRSV), which inhibits silencing by binding the siRNAs in a size‐specific manner (Silhavy *et al.*, 2002; Vargason *et al.*, 2003; Ye *et al.*, 2003) thereby inhibiting their incorporation into the RISC (Havelda *et al.*, 2003; Lakatos *et al.*, 2004).

The essential role of RNA silencing in the recovery phenotype was further demonstrated using mutant virus infection in which VSR was inactivated (Silhavy *et al.*, 2002; Szittya *et al.*, 2002). In these reports it was shown that the VSR‐inactivated virus initially caused severe symptoms, which gradually disappeared in the newly developed upper leaves. The viral RNA content of these leaves decreased below the detection level and they showed resistance against virus‐containing homologous sequences (Havelda *et al.*, 2003; Silhavy *et al.*, 2002; Szittya *et al.*, 2002). It is assumed that the virus causing the initial symptoms had activated viral RNA silencing. This inhibited the spread of virus infection into the upper leaves and caused them to be specifically immune to secondary infection of the same virus (Baulcombe, 2004; Szittya *et al.*, 2002). However, a recent report demonstrated the complexity of the plant antiviral response, showing that leaves recovered from viral symptoms contain infectious, replicating virus, but exhibit a loss of VSR activity as a result of overloading with a high amount of antiviral secondary siRNAs and this affected the viral symptoms (Kørner *et al.*, 2018).

Moreover, it turned out that although RNA silencing explains plant recovery from virus infection in specific virus–host combinations (Baulcombe, 2004; Burgyán and Havelda, 2011; Havelda *et al.*, 2003; Ratcliff *et al.*, 1997; Szittya *et al.*, 2002), this is not true for recovery in all plant–virus interactions. For example, the originally described recovery of ToRSV‐infected tobacco plants relies on the translational repression of the viral RNAs: it has been shown that *Nicotiana benthamiana* plants infected by ToRSV induced necrotic symptoms at the early stage of infection, which disappeared at a later stage although the level of viral RNA was not reduced in symptomless leaves (Ghoshal and Sanfaçon, 2015; Jovel *et al.*, 2007).

Plant recovery is often also associated with the exclusion of viruses from the plant SAM, which is one of the main plant meristems. The stem cells in these niches are maintained by a regulatory network. One of the main components of this network in the SAM is the homeodomain transcription factor WUSCHEL (WUS) and the CLAVATA (CLV) ligand‐receptor system (Brand *et al.*, 2000; Fletcher *et al.*, 1999; Schoof *et al.*, 2000). *WUS* is expressed in the organizing centre, the middle of the central zone of the meristem, and is required to induce and maintain the overlaying stem cells in an undifferentiated state (Mayer *et al.*, 1998). The loss of WUS function results in meristem collapse and differentiation caused by the lack of stem cell maintenance (Laux *et al.*, 1996). Another homeodomain transcription factor that acts in parallel with WUS in maintaining the meristem is SHOOT MERISTEMLESS (STM) (Long *et al.*, 1996). *STM* is expressed uniformly throughout the meristem and it stimulates cytokinin biosynthesis that inhibits differentiation (Andersen *et al.*, 2008; Yanai *et al.*, 2005).

There are hints and suggestions that virus exclusion from the meristem has a role in plant recovery and meristem exclusion has been attributed to RNA silencing mechanisms (Schwach *et al.*, 2005). A tobacco rattle virus (TRV) mutant deficient in the 16K protein, a weak suppressor of silencing, caused enhanced symptomatology on systemically infected leaves but failed to enter the meristem (Ghoshal and Sanfaçon, 2015; Martín‐Hernández and Baulcombe, 2008). It was suggested that meristem entry facilitated by the weak VSR and invasion of meristem is necessary to trigger antiviral systemic silencing. The movement of generated vsiRNAs leads to symptom recovery (Martín‐Hernández and Baulcombe, 2008). A similar role in transient meristem invasion was attributed to the cucumber mosaic virus (CMV) 2b VSR and this was also linked to the induction of symptom recovery (Mochizuki and Ohki, 2004; Sunpapao *et al.*, 2009). Moreover, a kinetic study demonstrated that the induction of RNA silencing occurred before the meristem entry of the invading virus and the RNA silencing was triggered by virus accumulation in infected leaves rather than by meristem invasion (Santovito *et al.*, 2014).

In line with this model, plants infected with viruses expressing VSRs often show tip necrosis, suggesting that the virus is able to invade the plant shoot, including the apical meristem, which finally leads to shoot necrosis and then plant death (Di Serio *et al*., 2010; Martín‐Hernández and Baulcombe, 2008; Schwach *et al.*, 2005). Indeed, a recent report showed that transgenic expression of RDR1, which likely enhances antiviral RNA silencing, inhibits severe symptom development by limiting the spread of the virus into the growing tips of infected plants, similarly to RDR6 (Lee *et al.*, 2016). Although these studies strongly suggest a key role of RNA silencing in virus exclusion from the plant SAM, direct evidence for the generalization of this model for all viruses is still missing.

To test whether antiviral silencing has a key role in meristem invasion, we infected *N. benthamiana* plants with CymRSV, which expresses the strong RNA silencing suppressor protein p19. Since the CymRSV‐induced shoot necrosis is quite fast, we also used the Cym19stop mutant virus (Dalmay *et al.*, 1993), which carries a stop codon in the p19 gene and cannot spread further than a few cell layers from the veins (Havelda *et al.*, 2003) (Fig. S1). We also infected transgenic *N. benthamiana* plants expressing a synthetic version of p19 VSR of CymRSV (p19syn plant) (Kontra *et al.*, 2016). p19syn plants express wild‐type (wt) p19 but the RNA sequence of the synthetic p19 gene has low homology with wt virus RNA to avoid transgene‐induced silencing against the Cym19stop virus. The p19 mutant virus can spread in these plants similarly to CymRSV in wt plants, although the process is a bit slower due to the lower amount of p19 (Kontra *et al.*, 2016) (Fig. S1). We performed *in situ* hybridization on the infected *N. benthamiana* shoots following the protocol previously described (Medzihradszky *et al*., 2014). Contrary to our expectations (based on previous studies with other viruses), we could not detect the CymRSV in any of the shoot meristems of virus‐infected plants, not even in the presence of an enhanced amount of p19 (p19syn plant infected with CymRSV) and at low temperature, which also inhibits RNA silencing (Fig. 1) (Szittya *et al.*, 2003). This experiment demonstrates that the inactivation of the antiviral silencing response by p19 VSR or low temperature is not enough to allow the replicating virus to invade the SAM. On the other hand, the suppression of virus‐induced antiviral silencing is crucial for the high accumulation of viral RNAs in the plant shoot (excluding the meristem) and for the development of fast shoot apical necrosis, as was previously demonstrated (Havelda *et al.*, 2003; Szittya *et al.*, 2002, 2003). To have a piece of more solid evidence, we also inoculated *DCL2/4*‐silenced plants (Cordero *et al.*, 2016) with Cym19stop virus. In this case, the viral p19 silencing suppressor and the antiviral silencing response of the host are both inactivated. The obtained results also show that the virus was excluded from the meristem (Fig. 1B). Apparently, it does not matter how we inactivate the host silencing machinery (either with a strong viral silencing suppressor or knocking down a host silencing component); the virus cannot invade the meristem. This finding further supports our hypothesis that RNA silencing is not the key factor in the viral meristem exclusion in this virus–host system.

**Figure 1:**
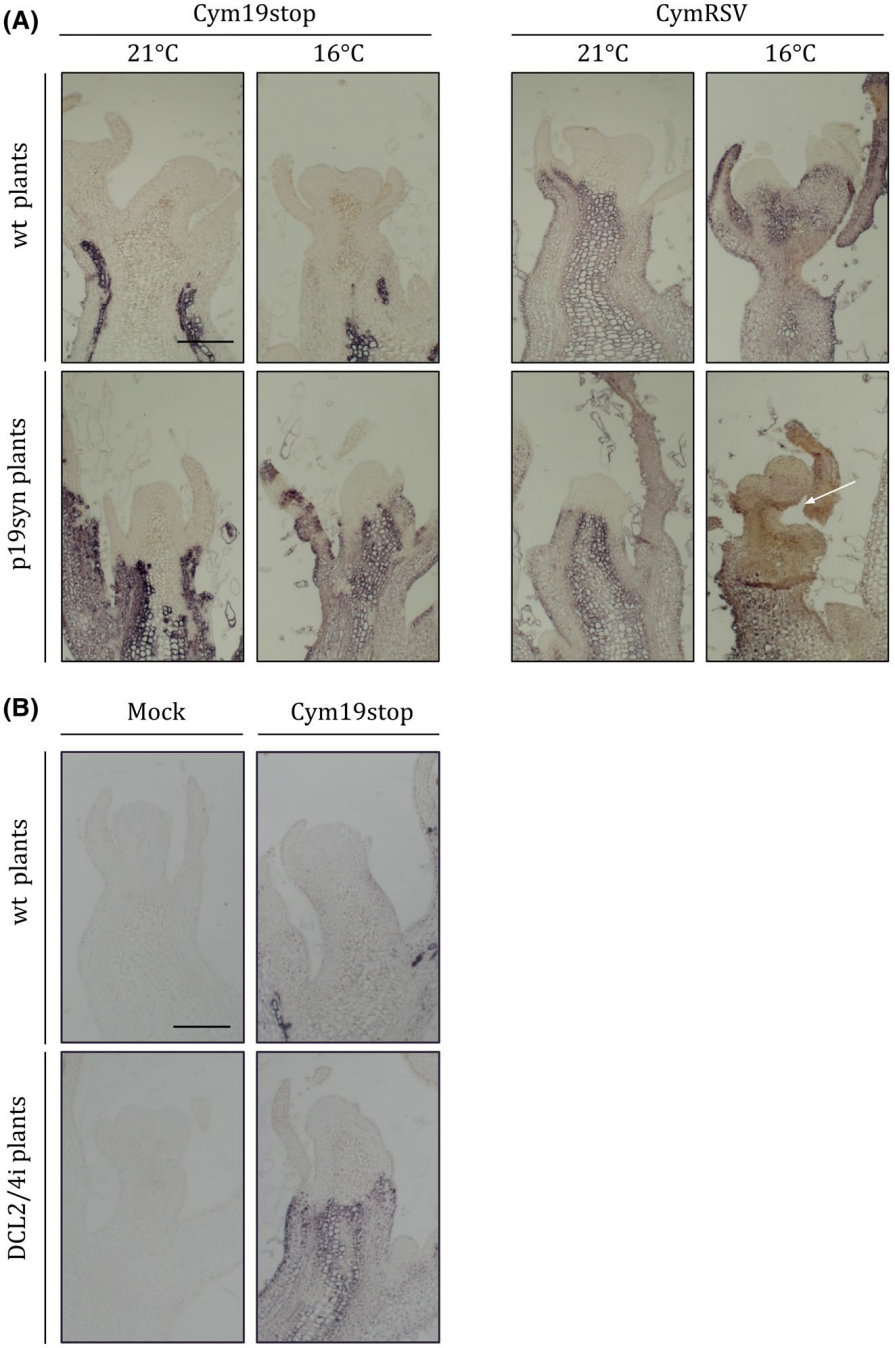
Detection of CymRSV in the shoot apical meristem (SAM) by *in situ* hybridization. Representative pictures of *in situ* hybridizations of consecutive longitudinal sections of infected meristems (*n* = 3–10) hybridized with CymRSV‐specific probes. Wild‐type *Nicotiana benthamiana* and transgenic plants expressing the p19 viral‐encoded silencing suppressor protein (VSR) of CymRSV (p19syn) (A) or *DCL2/4*‐silenced (DCL2/4i) plants (B) (Cordero *et al*., 2016) were infected either with wild‐type (wt) CymRSV or the VSR mutant Cym19stop as indicated. Plants were grown at 21 °C for 5 days (A and B) or at 16 °C for 9 days (A) after inoculation. Arrow indicates necrotic tissue. Bars = 200 µm.

To have a better view of the spatiotemporal progression of infection and the transcriptional changes in virus‐infected plant shoot apices, we performed a series of *in situ* hybridizations (Fig. 2). This experiment showed that after 3 days post‐inoculation (dpi) the virus is already present in the shoot, but the endogenous transcription of the housekeeping *GLYCERALDEHYDE 3-PHOSPHATE DEHYDROGENASE* (*GAPDH*) and the *PATHOGENESIS-RELATED PROTEIN Q* (*PRQ*) (Li *et al*., 2018b; Pesti *et al.*, 2019) are unchanged. In contrast, after 4 dpi, in the virus‐invaded plant tissues close to the SAM, the *GAPDH* level is undetectable, while *PRQ* is greatly induced. It is worth noting that GAPDH is essential for the replication of the invading virus (Havelda *et al.*, 2008; Wang and Nagy, 2008) and lack of GAPDH stops the virus replication and spread. Although the wt virus was clearly excluded from the SAM, it was able to induce shoot necrosis of the plant. More importantly, the virus infection specifically inhibited the expression of SAM‐specific genes such as *WUS*, the central player of the meristem‐specific regulatory network (Fig. 3). One would expect that the loss of *WUS* results in meristem collapse, and the high accumulation of virus close to the meristem leads to tip necrosis. However, meristem collapse alone does not induce shoot necrosis, as was observed using the *wus‐1* loss‐of‐function mutant (Laux *et al.*, 1996).

**Figure 2:**
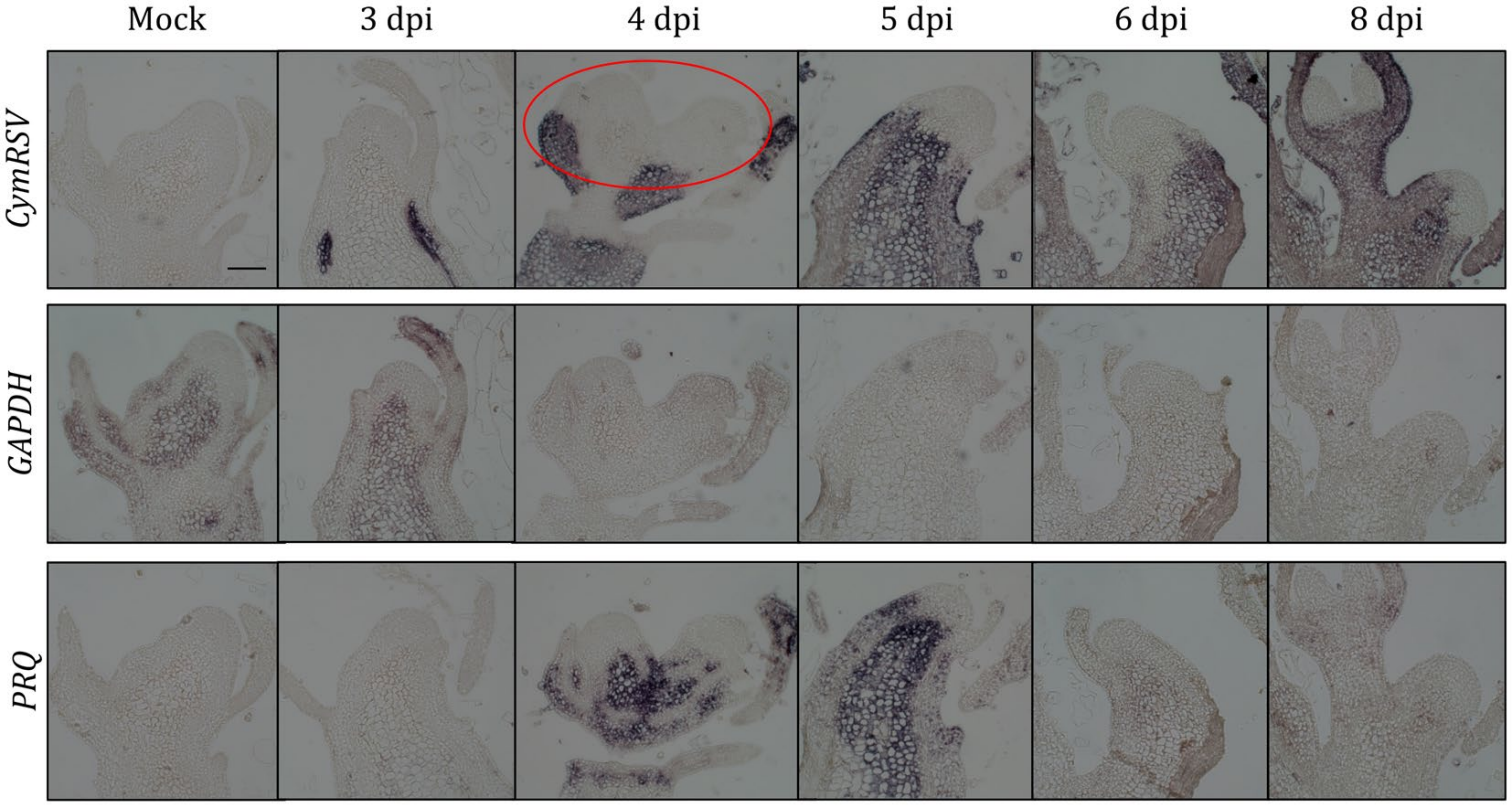
Monitoring the movement of CymRSV in shoot apices in time. *In situ* hybridization of longitudinal sections of mock‐treated or CymRSV‐infected shoot apical meristems 3 to 8 days post‐inoculation (dpi), as indicated. Sections were hybridized with CymRSV‐ or gene‐specific probes to detect *GLYCERALDEHYDE 3‐PHOSPHATE DEHYDROGENASE A subunit 2* (*GAPDH*), *PATHOGENESIS‐RELATED PROTEIN Q* (*PRQ*). The red circle indicates the tissue collected for the RNA‐Seq experiments. Bar = 100 µm.

**Figure 3:**
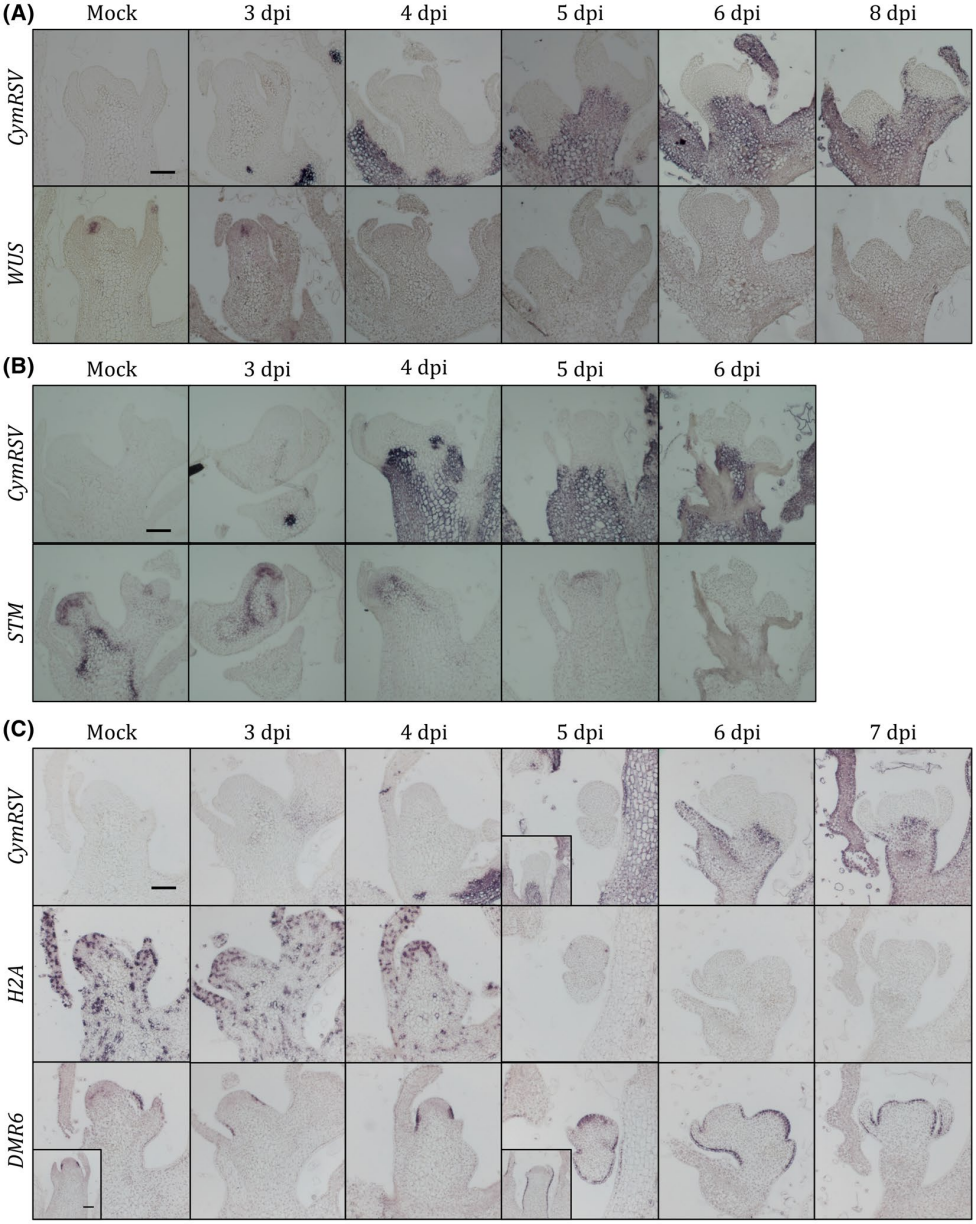
Expression pattern of selected genes in CymRSV‐infected shoot apices. *In situ* hybridization of longitudinal sections of mock and CymRSV‐infected plants hybridized with CymRSV‐ or gene‐specific probes as indicated on the left side: (A) *WUSCHEL* (*WUS*), (B) *SHOOT MERISTEMLESS* (*STM*), (C) *HISTONE 2A* (*H2A*) and *DOWNY MILDEW RESISTANT 6* (*DMR6*). Note that meristems in (C) are from a different infection and embedding that can cause different timing (days post‐inoculation, dpi) in the expression changes. Bars = 100 µm.

The above *in situ* data suggest that there is a sharp change in the expression patterns from 3 to 4 dpi. Thus, to capture the genome‐wide gene expression changes caused by the advancing virus infection, we collected meristem tissue samples from 4 dpi mock‐ and CymRSV‐infected plants in four biological replicates (45 meristems for each replicate) for transcriptome analysis. RNA extraction was performed as described previously (Burgyán *et al.*, 2000). It is important to note that these samples contained not only meristem cells but tissues close to the meristem, as indicated in Fig. 2. Using the purified RNAs, we prepared polyA‐selected, unstranded sequencing libraries and sequenced them on an Illumina HiSeq 2000 platform with a 75 bp single‐end chemistry. The alignment of the sequences to the *N. benthamiana* v. 1.0.1 reference transcriptome and the differential expression analysis was carried out with kallisto and sleuth, respectively (Pimentel *et al.*, 2017). For the criteria considering a transcript as differentially expressed see the legend of Fig. S2. Applying the filtering rules we identified 5747 significantly up‐regulated and 4369 down‐regulated transcripts (Fig. S2 and Table S1A,B). We checked the expression pattern of some marker genes and potentially meristem‐specific genes in the sequencing data (Fig. S3). We verified the results of the sequencing data by RT‐qPCR (Fig. S4). We also checked the expression of the known meristem‐specific genes (Fig. S5 and Table S1C). To characterize the transcriptional changes functionally, we performed Gene Ontology (GO)‐term enrichment analysis of the significantly changed genes using PlantRegMap (Jin *et al.*, 2017) and REVIGO (Supek *et al.*, 2011). Analysing the results in detail, we can conclude that the up‐regulated genes are mainly related to cell defence (plasma membrane‐located receptor kinases, hypersensitive response, salicylic acid and ethylene signalling, programmed cell death; Fig. 4 and Table S2A), while the down‐regulated genes are related to DNA replication and organization, shoot meristem development and plasmodesmata function (Fig. 4 and Table S2B).

**Figure 4:**
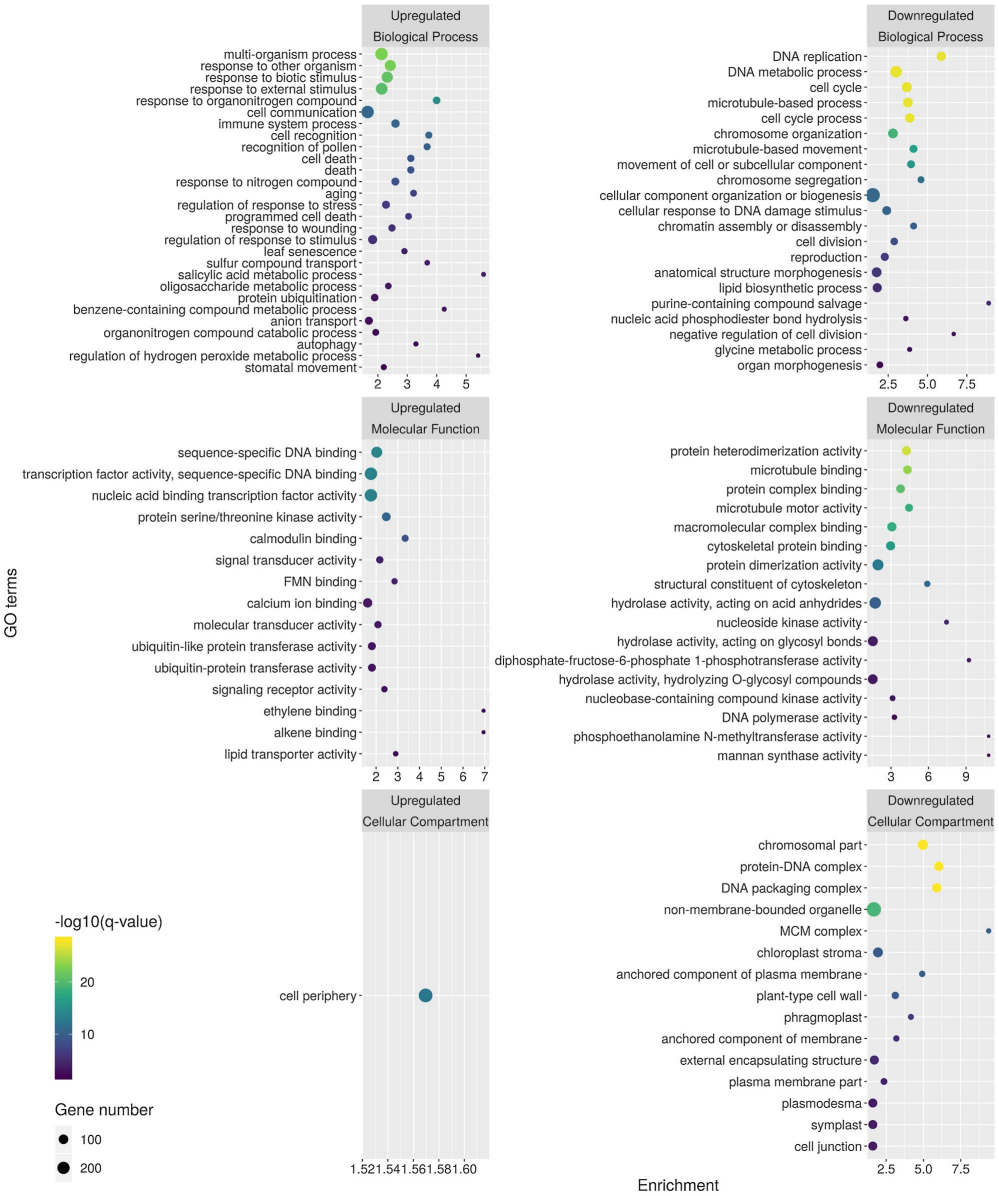
Significantly enriched GO categories among the up‐ and down‐regulated genes. The differential expression analysis was performed with sleuth (Pimentel *et al*., 2017). For the criteria considering a gene as differentially expressed see Fig. S2. We performed the GO‐term enrichment analysis using PlantRegMap (Jin *et al*., 2017) and REVIGO (Supek *et al*., 2011). Enrichment of GO terms was calculated by dividing the number of the observed term in the query list with the expected number of that particular term. Only terms with an enrichment greater than 1.5 and a *q*‐value less than 0.01 are shown. The result of the GO‐term enrichment analysis is in Table S2.

Although many transcripts were up‐regulated on CymRSV infection, most of the already known meristem‐regulating genes were down‐regulated in our dataset (Fig. S5 and Table S1C). To explore how well the transcriptome analysis captured the changes in the meristem and to distinguish these changes from those in the infected tissue part, we performed *in situ* hybridization with a few selected genes (Fig. 3 and Table S3). According to this experiment, *WUSCHEL (WUS)*, which is essential to maintain the meristem, was not detectable in the organizing centre after 4 dpi (Fig. 3). The level of *SHOOT MERISTEMLESS (STM)* also decreased (Fig. 3), indicating that the meristem function is disturbed. *HISTONE 2A* (*H2A*) was also strongly down‐regulated in the infected shoots (Fig. 3C). In a recent study, down‐regulation of *H2B* was implicated in the salicylic acid‐mediated defence against potato virus X infection (Yang *et al.*, 2019).

Since even the stress‐related *PRQ* was up‐regulated only in the infected tissues and not detectable in the meristem, we wondered if we would find any genes up‐regulated in the meristem. For this purpose, we selected a few genes showing up‐regulation in our RNA‐Seq data (Fig. S3 and Table S3) and performed *in situ* hybridizations with them. This showed that most of the selected up‐regulated genes expressed at a higher level only in the infected tissue (Fig. S6). However, *DOWNY MILDEW RESISTANT 6* (*DMR6*), which is a salicylic acid 5‐hydroxylase, was strongly expressed in the peripheral zone of the meristem but only in the uninfected meristem cells (Fig. 3C).

These data suggest that the meristem is not functional anymore and that these changes are likely programmed as a consequence of the accumulated virus in the tissues around the meristem. We wondered if the down‐regulation of *WUS* has any connection to the shut‐off phenomenon when the down‐regulation of host gene expression is associated with the virus replication (Wang and Maule, 1995). To test this, we extended our experiments with turnip vein‐clearing virus (TVCV) (Csorba *et al.*, 2007) and cucumber mosaic virus (CMV) infected samples. TVCV causes shut‐off (Havelda *et al.*, 2008; Pesti *et al.*, 2019) and similar symptoms (shoot necrosis) as the CymRSV infection. We got similar results as with CymRSV except for *WUS*, which was unchanged compared to mock even after 6 dpi (Figs 5 and S7). CMV infection does not cause shut‐off and results in weak symptoms. Importantly, in these samples the *WUS* expression stays on, similarly to the TVCV infection*,* and, as expected, the *GAPDH*, *H2A* and *STM* expressions were also unchanged (Fig. S8). Interestingly, the *PRQ* expression is up‐regulated at 4 dpi but then decreases again. Taken together, these results show that the down‐regulation of *WUS* is specific to CymRSV infection and is not the consequence of the activated shut‐off. Moreover, there is no direct connection between the down‐regulation of *WUS* and the shoot necrosis in general, since TVCV necrotized the shoot while CMV did not.

**Figure 5:**
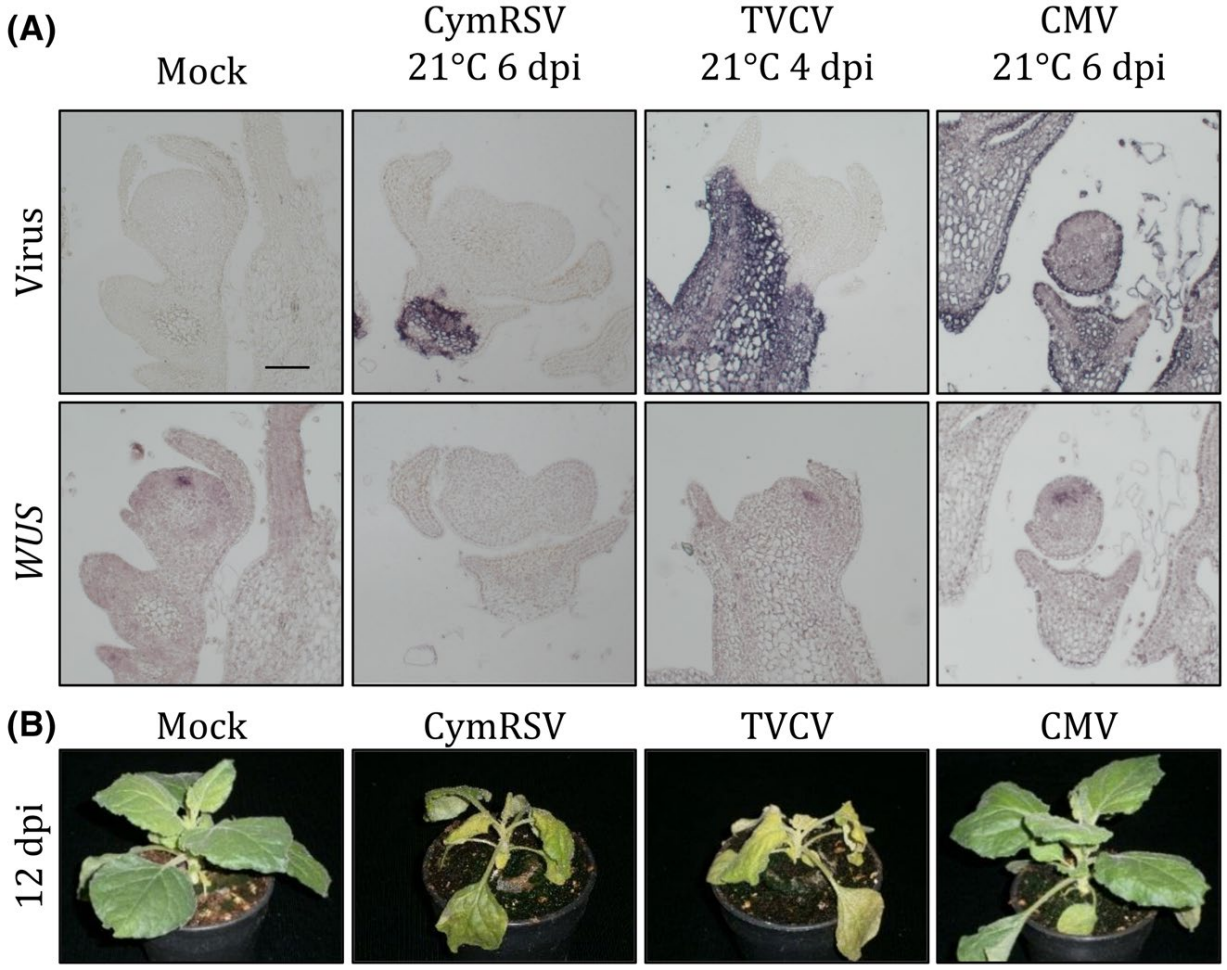
*WUS* expression and viral symptoms of plants infected with three different viruses. (A) *In situ* hybridization of consecutive longitudinal sections of mock‐, CymRSV‐, TVCV‐ and CMV‐infected shoot apical meristems hybridized with probes to detect the virus or *WUSCHEL* (*WUS*). Bar = 100 µm. (B) Symptoms of mock‐, CymRSV‐, TVCV‐ or CMV‐infected *Nicotiana benthamiana* plants 12 days after infection.

Our results clearly demonstrate that in a compatible interaction between a virus and its host plant, the virus infection causes drastic alterations in the plant transcriptome, leading to symptom development. In many cases, symptom attenuation can be seen in the upper part of the plant, called recovery (Ghoshal and Sanfaçon, 2015). The capability for recovery depends on the host plant species/variety–virus interaction (Bengyella *et al.*, 2015; Paudel and Sanfaçon, 2018) and is influenced by environmental factors, such as temperature (Fondong *et al.*, 2000). Recovery is also linked to gene silencing and its dependence on temperature (Szittya *et al.*, 2003). In the reported experiments, the infected plants were grown at various temperatures after infection. At 27 °C, the virus could not invade the whole leaf blade or spread close to the meristem, leading to recovering from the infection. Here we tested whether the plants can recover at 27 °C after they developed symptoms at 21 °C. We found that most of the plants could recover but only from the axillary buds (Fig. S9), showing that the reprogramming of the transcriptome is not reversible by temperature and leads to the loss of SAM function. Our data also suggest that the loss of meristem function is not because of lost *WUS* expression, but might be a result of a reprogrammed hormonal network.

It is worth noting that the expression of three E3 SUMO‐protein ligase *SIZ1* homologues is up‐regulated (Fig. S10). *SIZ1* is a regulator of salicylic acid signalling, immunity and shoot development (Hammoudi *et al.*, 2018; Lee *et al.*, 2007; Miura *et al.*, 2010; Niu *et al.*, 2019), making it a good candidate as a silencing‐independent factor of virus‐induced meristem shut‐down. Recently, an autophagy‐related gene, *Beclin‐1* (*BECN1*, *ATG6*), was shown to be induced by turnip mosaic virus (TuMV) infection and to restrict viral replication in *N. benthamiana* plants by binding to the viral replicase protein (Li *et al*., 2018a, p. 1). *Beclin‐1* was also induced in our samples, along with other autophagy‐related genes (Fig. S11), which suggests that this mechanism of viral restriction is not TuMV‐specific and that virus‐induced autophagy is a common response to virus infection. In a comparative transcriptome analysis of chrysanthemum infected with three different viruses, the authors identified 33 differentially expressed genes that were common between the three viral infections (Choi *et al.*, 2015). Notably, members of the minichromosome maintenance complex were found to be down‐regulated, just like in our dataset (Figs 4 and S12). This suggests that functions related to DNA replication and cell division are usually corrupted during viral infection, which might lead to genome instability and programmed cell death.

Taken together, our results highlight the high variability of plant strategies to counteract invading viral pathogens.

## Supporting information


**Fig. S1** Testing the CymRSV probe. *In situ *hybridization of cross‐sections of mock, Cym19stop‐, and CymRSV‐infected plant leaves using an RNA probe against CymRSV. Bars = 200 µm.

**Fig. S2** Exploratory analysis of the RNA‐Seq data. (A) Principal component analysis of the log_2_‐transformed, normalized transcript expressions (TPM) in the samples. We prepared four biological replicates per treatment. The first component explains more than two‐thirds of the variances in the expression values and separates the mock and the virus‐infected samples. (B) MA‐plot showing the biased estimator of the fold‐changes (the beta coefficient in the sleuth model) in the transcript expressions as a function of the mean transcript abundance (counts). The values shown in the axes are the exponentials of the original log‐transformed values on a log_10_ scale. The positive beta values represent increased, while the negative values represent decreased transcript expression in the virus‐infected samples. For the differential expression analysis, the *P*‐values were calculated using the Wald test and corrected for multiple testing to get the *Q*‐values according to the Benjamini–Hochberg method at a false discovery rate of 1%. We considered a transcript differentially expressed if its mean abundance across the samples was at least 10 (vertical dashed line), the absolute value of its beta was at least 2 (horizontal dashed line), and its *Q*‐value was less than 0.01. The red dots mark the transcripts that passed these filters. The numbers after the upward and the downward arrow denote the number of the up‐ and the down‐regulated transcripts, respectively.

**Fig. S3** Expression of selected genes that were analysed by *in situ* hybridization experiments. The normalized expression values (transcript per million, TPM) of the indicated genes were estimated by kallisto (Pimentel *et al.*, 2017). The boxplots represent the summary of the bootstrap analysis which estimates the technical variances in a sample. Four biological replicates were prepared (Mock 1–4, Virus 1–4). The *WUSCHEL* (*WUS*) and the *SHOOT MERISTEMLESS* (*STM*) gene strictly expresses in the meristem, while the *GLYCERALDEHYDE‐PHOSPHATE DEHYDROGENASE* (*GAPDH*) is an enzyme that is required for virus replication. The *PATHOGENESIS‐RELATED PROTEIN Q* (*PRQ*) is a well‐known stress‐induced marker gene. *HISTONE 2A* (*H2A*), *DOWNY MILDEW RESISTANT 6* (*DMR6*) is a salicylic acid 5‐hydroxylase, *UDP‐GLYCOSYLTRANSFERASE 74 F1* (*UGT74F1*) transfers UDP:glucose to salicylic acid, *CYTOCHROME P450* (*CYP450*) is a hydroxylase involved in the metabolism of many bioactive compounds, including hormones, while *CALMODULIN‐BINDING PROTEIN* (CaM‐binding) is a component of many stress‐related signalling pathways.

**Fig. S4** Validation of the high‐throughput sequencing results by quantitative real‐time RT‐PCR. Some of the investigated genes shown in Fig. S3 was also measured by RT‐qPCR. Gene levels were normalized to the *SEC23* and *PRX2B* (Table S3) reference gene levels in the same sample. Reference genes were selected from the least significantly changed genes according to the RNA‐Seq analysis, with moderate (~50 and ~600 TPM) mean expression levels and minimal variation of expression levels across all the samples. The values are means of three independent experiments with SE. An unpaired, one‐tailed *t*‐test was performed to estimate statistical significance. ***, *P* ≤ 0.001; **, *P* ≤ 0.01; *, *P* ≤ 0.05, ns = not significant.

**Fig. S5** Expression of meristem‐specific genes. The normalized expression values (transcript per million, TPM) of the indicated genes were estimated by kallisto (Pimentel *et al.*, 2017), log_2_‐transformed and *z*‐scores were calculated. The *z*‐scores show how many standard deviations the given value is above (red) or below (blue) from the mean (white) of all the values in the row. We show the values of the four biological replicates (Mock 1–4, Virus 1–4).

**Fig. S6** Expression pattern of selected genes in CymRSV‐infected shoot apices. *In situ *hybridization of longitudinal sections of shoot apices of mock‐ and CymRSV‐infected plants 4 or 5 dpi, hybridized with CymRSV‐ or gene‐specific probes as indicated: *UDP‐GLYCOSYLTRANSFERASE 74 F1* (*UGT74F1*), *CYTOCHROME P450* *SUPERFAMILY PROTEIN* (*CYP450*), or Calmodulin‐binding (CaM‐BP). Bars = 100 µm.

**Fig. S7** *In situ *hybridization of TVCV‐infected shoot apices. *In situ *hybridization of longitudinal sections of mock‐ and TVCV‐infected plants 4 or 5 dpi hybridized with a virus‐ or gene‐specific probes as indicated: *GLYCERALDEHYDE 3‐PHOSPHATE DEHYDROGENASE A SUBUNIT 2* (*GAPDH*), *PATHOGENESIS‐RELATED PROTEIN Q *(*PRQ*), *WUSCHEL* (*WUS*), *SHOOT MERISTEMLESS* (*STM*). Bars = 100 µm.

**Fig. S8** *In situ *hybridization of CMV‐infected shoot apices. *In situ *hybridization of longitudinal sections of mock‐ and CMV‐infected plants hybridized with a virus‐ or gene‐specific probes as indicated on the left side: *GLYCERALDEHYDE 3‐PHOSPHATE DEHYDROGENASE A SUBUNIT 2* (*GAPDH*), *PATHOGENESIS‐RELATED PROTEIN Q* (*PRQ*), *HISTONE 2A* (*H2A*), *WUSCHEL* (*WUS*), *SHOOT MERISTEMLESS* (*STM*). Bars = 100 µm.

**Fig. S9** Recovery from side shoots. Symptoms of mock and CymRSV‐infected *Nicotiana benthamiana *plants grown at 21 °C 4–8 days after infection, then transferred to 27 °C for 3 weeks. Symptoms of a representative mock‐ and CymRSV‐infected plant grown at 21 °C one week after infection (upper picture) and at 27 °C four weeks after infection (lower picture).

**Fig. S10** Expression of the significantly up‐regulated *SIZ1* homologue genes in the SAM region. The normalized expression values (transcript per million, TPM) of the indicated genes were estimated by kallisto (Pimentel *et al.*, 2017), log_2_‐transformed and *z*‐scores were calculated. The *z*‐scores show how many standard deviations the given value is above (red) or below (blue) from the mean (white) of all the values in the row. We show the values of the four biological replicates (Mock 1–4, Virus 1–4). For criteria of the significantly changed expressions see legend of Fig. S2.

**Fig. S11** Expression of the significantly up‐regulated autophagy‐related genes in the SAM region. The normalized expression values (transcript per million, TPM) of the indicated genes were estimated by kallisto (Pimentel *et al.*, 2017), log_2_‐transformed and *z*‐scores were calculated. The *z*‐scores show how many standard deviations the given value is above (red) or below (blue) from the mean (white) of all the values in the row. We show the values of the four biological replicates (Mock 1–4, Virus 1–4). For criteria of the significantly changed expressions see legend of Fig. S2.

**Fig. S12** Expression of the significantly down‐regulated MCM‐complex genes in the SAM region. The normalized expression values (transcript per million, TPM) of the indicated genes were estimated by kallisto (Pimentel *et al.*, 2017), log_2_‐transformed and *z*‐scores were calculated. The *z*‐scores show how many standard deviations the given value is above (red) or below (blue) from the mean (white) of all the values in the row. We show the values of the four biological replicates (Mock 1–4, Virus 1–4). For criteria of the significantly changed expressions see legend of Fig. S2.

**Table S1 **RNA‐Seq analysis. The annotated expression tables and the result of the differential expression analysis for the up‐regulated (Table S1A) and down‐regulated transcripts (Table S1B), the meristem‐specific genes (Table S1C), and all the tested transcripts (Table S1D).

**Table S2 **GO‐term enrichment analysis. The result of the GO‐term enrichment analysis without the simplification by REViGO. Significantly enriched GO‐terms among the up‐regulated (Table S2A) and down‐regulated (Table S2B) genes.

**Table S3** Primers and probes. Information about the primers and probes used in this study.

## Data Availability

The data that support the findings of this study are openly available in the Gene Expression Omnibus (GEO) database at www.ncbi.nlm.nih.gov/geo, accession number GSE131476.
