# Supplementary figures and images for: Transcriptome reprogramming in the shoot apical meristem of CymRSV‐infected Nicotiana benthamiana plants associates with viral exclusion and the lack of recovery
Source: Mol Plant Pathol. 2019 Sep 27;20(12):1748–58. doi: 10.1111/mpp.12875 (PMC6859499; doi:10.1111/mpp.12875)

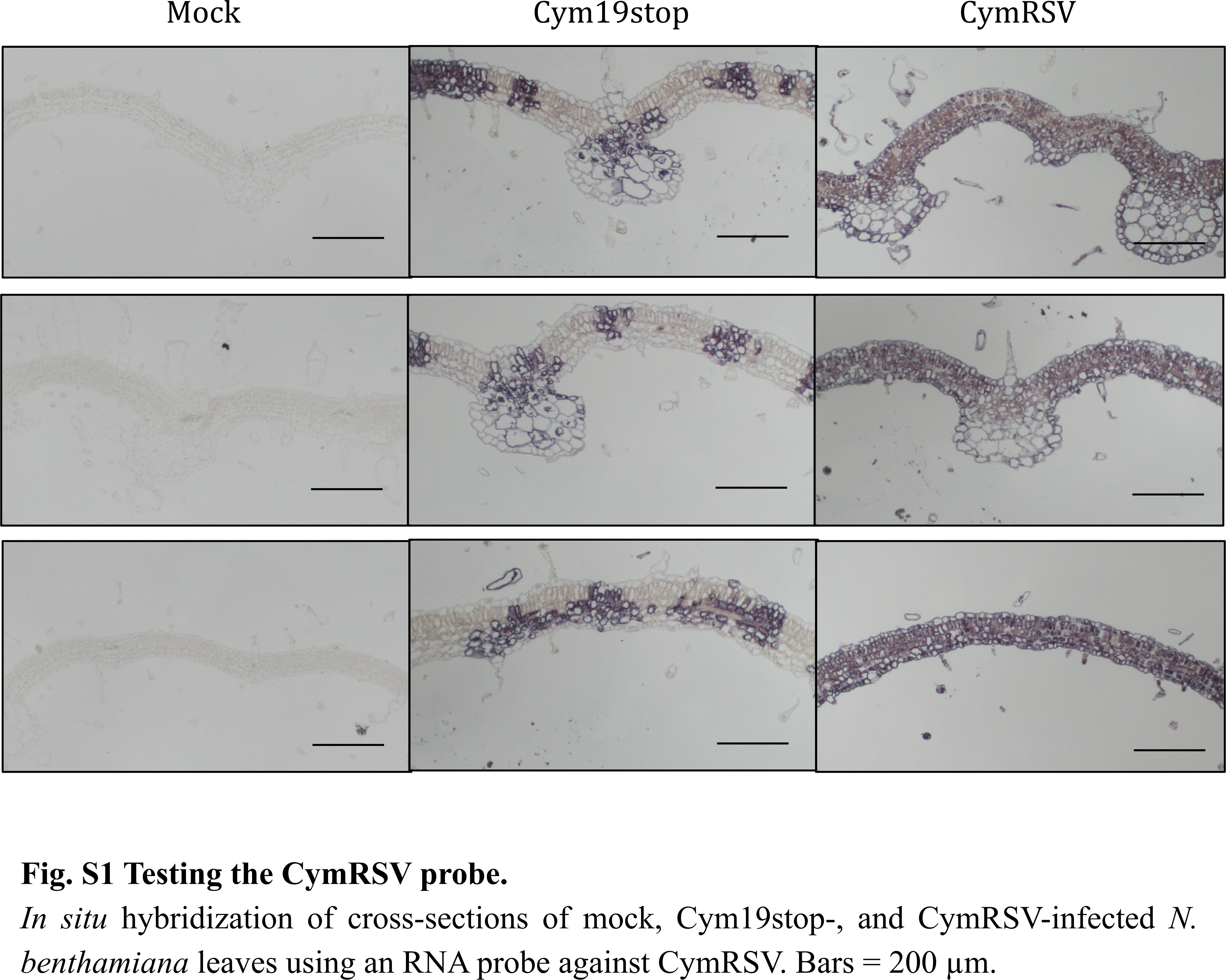

Supplement: Supplementary file 1 — Fig. S1 Testing the CymRSV probe. In situ hybridization of cross‐sections of mock, Cym19stop‐, and CymRSV‐infected plant leaves using an RNA probe against CymRSV. Bars = 200 µm. [file MPP-20-1748-s001.jpg]

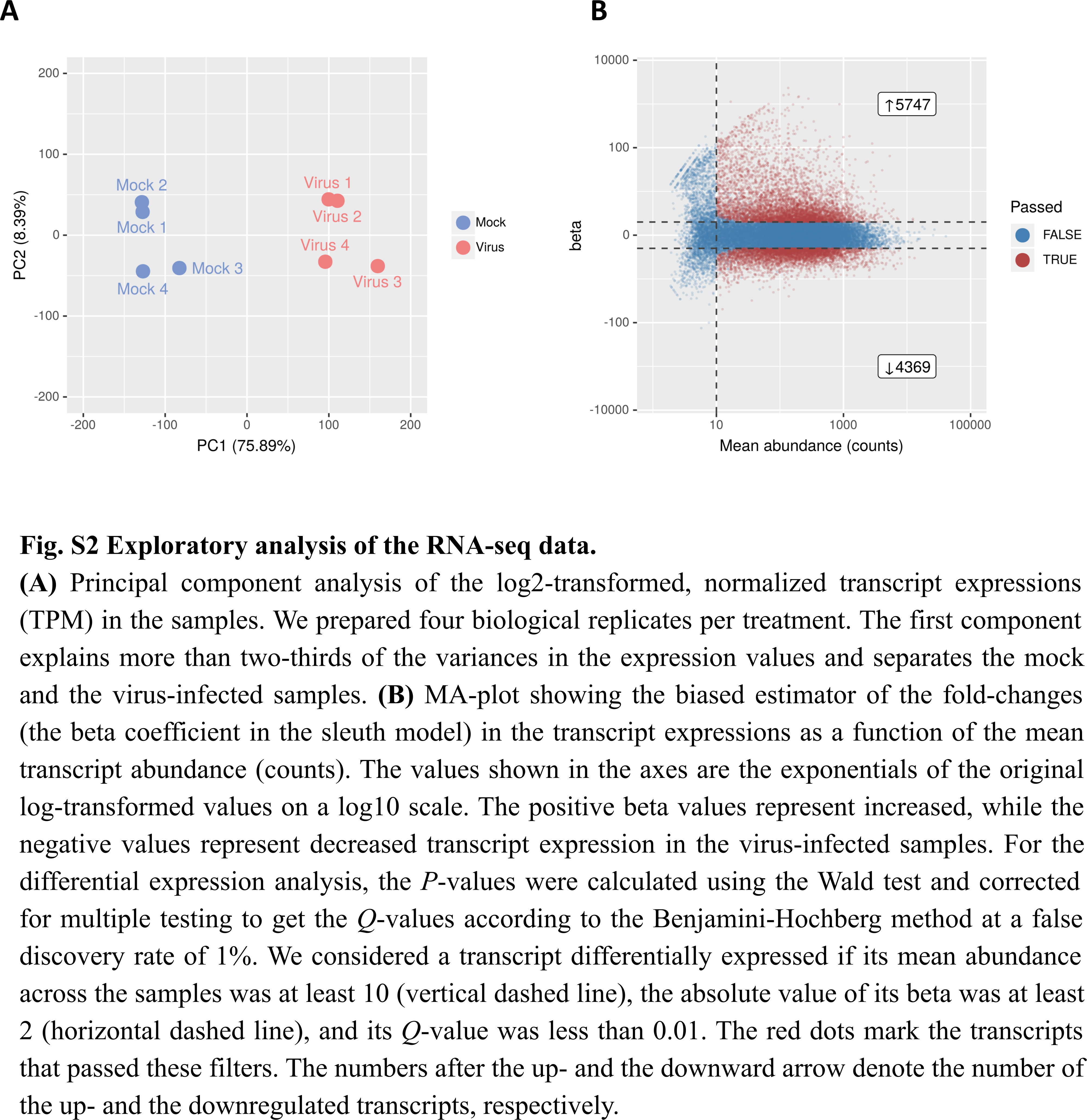

Supplement: Supplementary file 2 — Fig. S2 Exploratory analysis of the RNA‐Seq data. (A) Principal component analysis of the log2‐transformed, normalized transcript expressions (TPM) in the samples. We prepared four biological replicates per treatment. The first component explains more than two‐thirds of the variances in the expression values and separates the mock and the virus‐infected samples. (B) MA‐plot showing the biased estimator of the fold‐changes (the beta coefficient in the sleuth model) in the transcript expressions as a function of the mean transcript abundance (counts). The values shown in the axes are the exponentials of the original log‐transformed values on a log10 scale. The positive beta values represent increased, while the negative values represent decreased transcript expression in the virus‐infected samples. For the differential expression analysis, the P‐values were calculated using the Wald test and corrected for multiple testing to get the Q‐values according to the Benjamini–Hochberg method at a false discovery rate of 1%. We considered a transcript differentially expressed if its mean abundance across the samples was at least 10 (vertical dashed line), the absolute value of its beta was at least 2 (horizontal dashed line), and its Q‐value was less than 0.01. The red dots mark the transcripts that passed these filters. The numbers after the upward and the downward arrow denote the number of the up‐ and the down‐regulated transcripts, respectively. [file MPP-20-1748-s002.jpg]

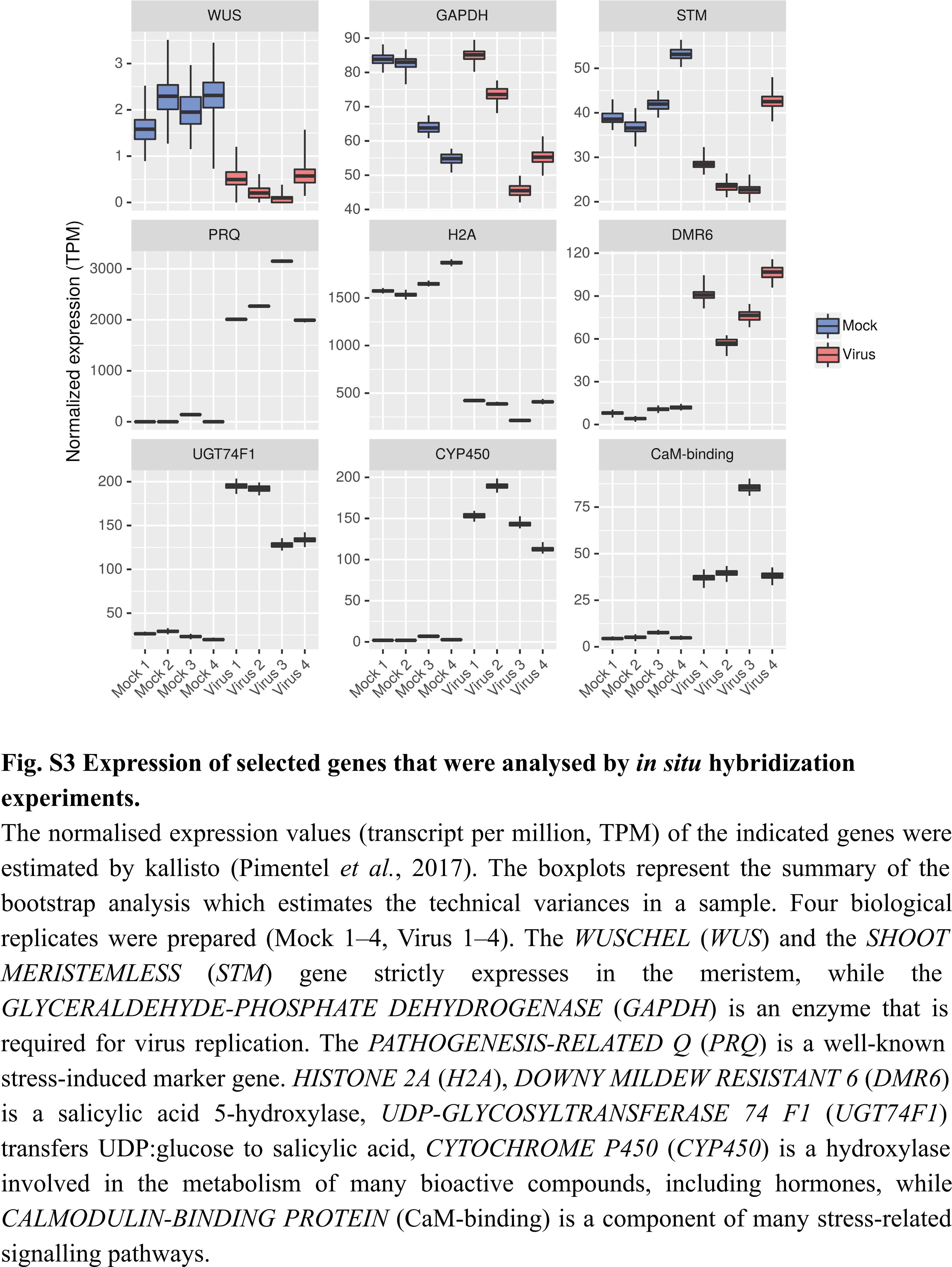

Supplement: Supplementary file 3 — Fig. S3 Expression of selected genes that were analysed by in situ hybridization experiments. The normalized expression values (transcript per million, TPM) of the indicated genes were estimated by kallisto (Pimentel et al., 2017). The boxplots represent the summary of the bootstrap analysis which estimates the technical variances in a sample. Four biological replicates were prepared (Mock 1–4, Virus 1–4). The WUSCHEL (WUS) and the SHOOT MERISTEMLESS (STM) gene strictly expresses in the meristem, while the GLYCERALDEHYDE‐PHOSPHATE DEHYDROGENASE (GAPDH) is an enzyme that is required for virus replication. The PATHOGENESIS‐RELATED PROTEIN Q (PRQ) is a well‐known stress‐induced marker gene. HISTONE 2A (H2A), DOWNY MILDEW RESISTANT 6 (DMR6) is a salicylic acid 5‐hydroxylase, UDP‐GLYCOSYLTRANSFERASE 74 F1 (UGT74F1) transfers UDP:glucose to salicylic acid, CYTOCHROME P450 (CYP450) is a hydroxylase involved in the metabolism of many bioactive compounds, including hormones, while CALMODULIN‐BINDING PROTEIN (CaM‐binding) is a component of many stress‐related signalling pathways. [file MPP-20-1748-s003.jpg]

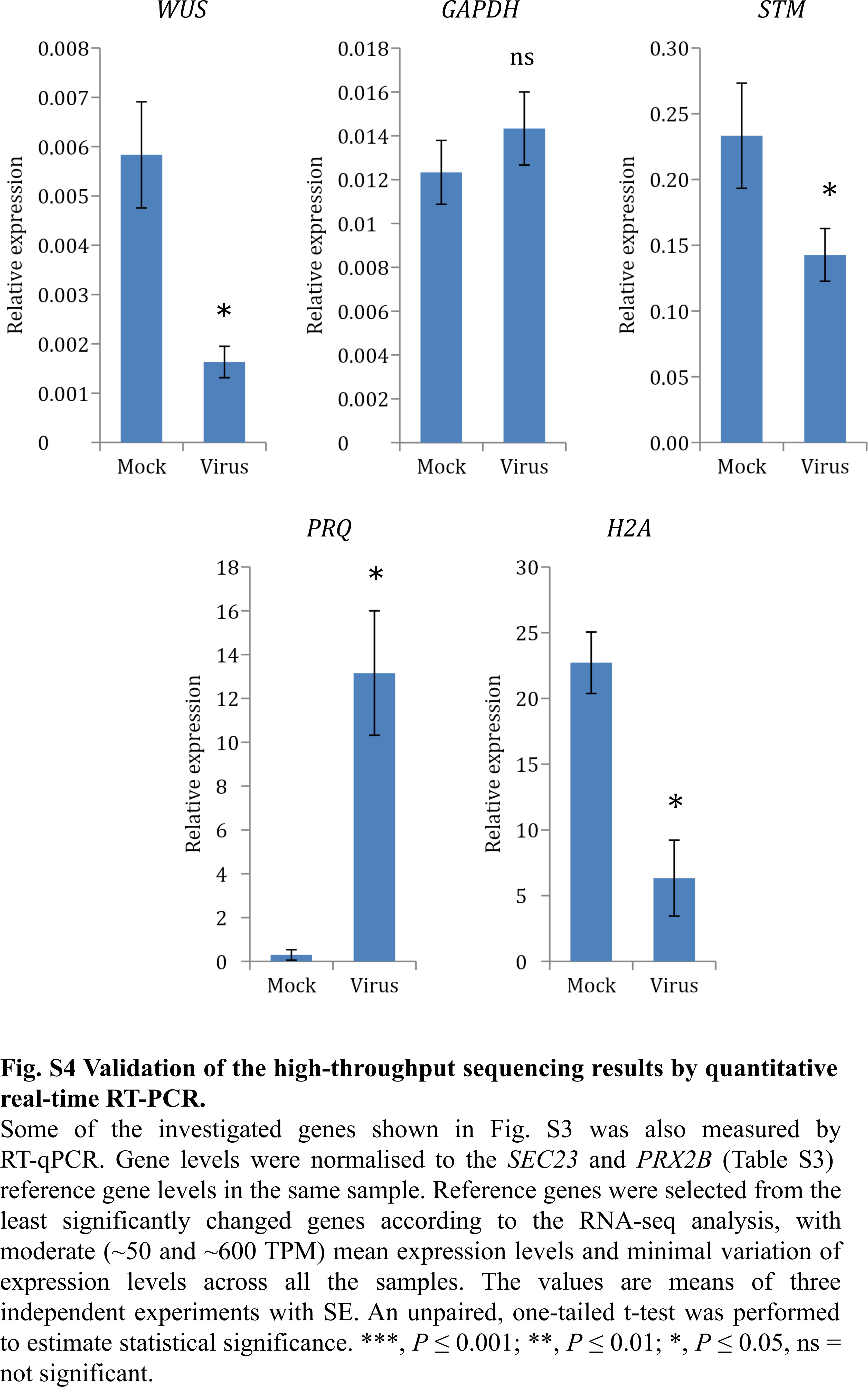

Supplement: Supplementary file 4 — Fig. S4 Validation of the high‐throughput sequencing results by quantitative real‐time RT‐PCR. Some of the investigated genes shown in Fig. S3 was also measured by RT‐qPCR. Gene levels were normalized to the SEC23 and PRX2B (Table S3) reference gene levels in the same sample. Reference genes were selected from the least significantly changed genes according to the RNA‐Seq analysis, with moderate (~50 and ~600 TPM) mean expression levels and minimal variation of expression levels across all the samples. The values are means of three independent experiments with SE. An unpaired, one‐tailed t‐test was performed to estimate statistical significance. ***, P ≤ 0.001; **, P ≤ 0.01; *, P ≤ 0.05, ns = not significant. [file MPP-20-1748-s004.jpg]

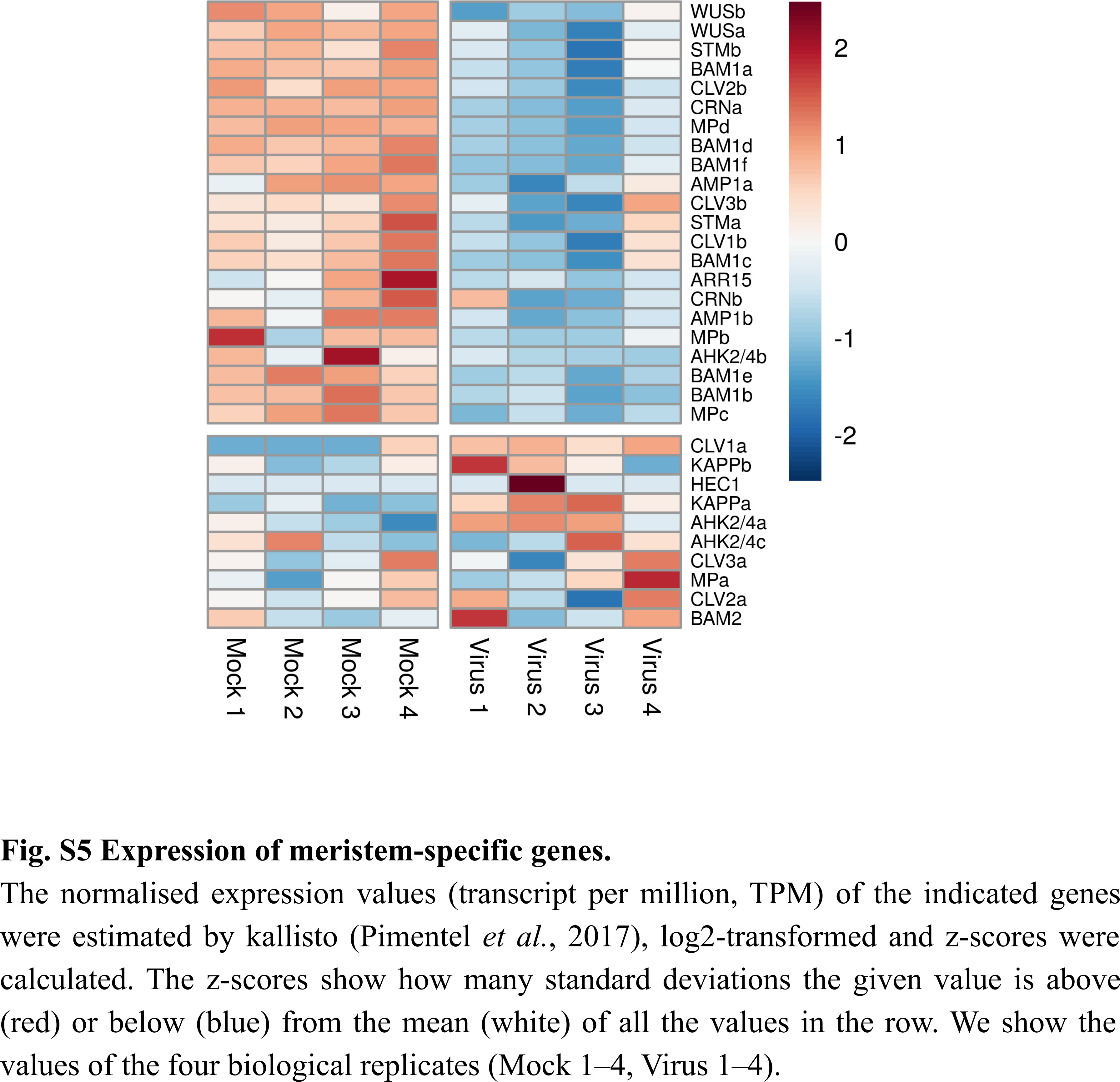

Supplement: Supplementary file 5 — Fig. S5 Expression of meristem‐specific genes. The normalized expression values (transcript per million, TPM) of the indicated genes were estimated by kallisto (Pimentel et al., 2017), log2‐transformed and z‐scores were calculated. The z‐scores show how many standard deviations the given value is above (red) or below (blue) from the mean (white) of all the values in the row. We show the values of the four biological replicates (Mock 1–4, Virus 1–4). [file MPP-20-1748-s005.jpg]

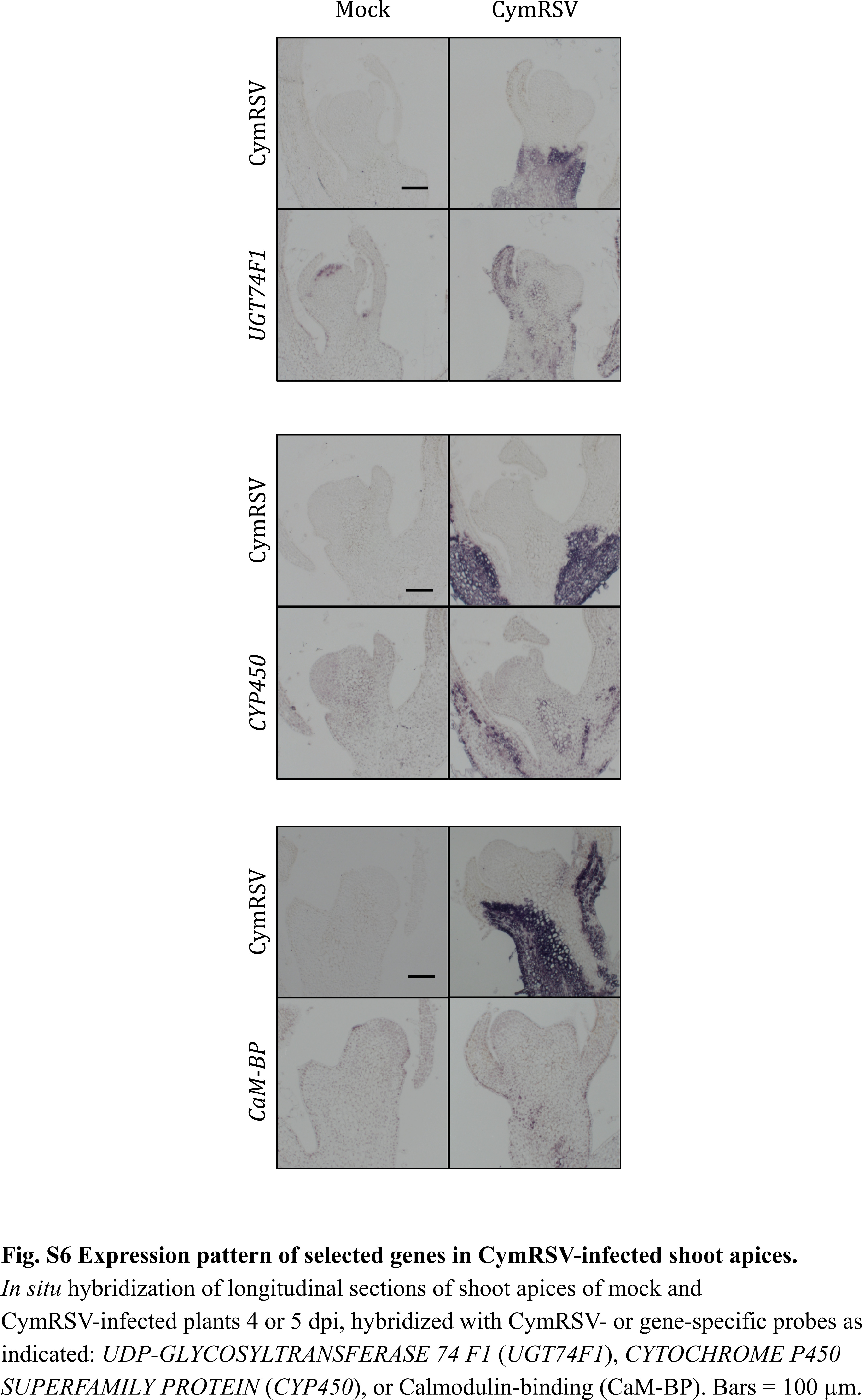

Supplement: Supplementary file 6 — Fig. S6 Expression pattern of selected genes in CymRSV‐infected shoot apices. In situ hybridization of longitudinal sections of shoot apices of mock‐ and CymRSV‐infected plants 4 or 5 dpi, hybridized with CymRSV‐ or gene‐specific probes as indicated: UDP‐GLYCOSYLTRANSFERASE 74 F1 (UGT74F1), CYTOCHROME P450 SUPERFAMILY PROTEIN (CYP450), or Calmodulin‐binding (CaM‐BP). Bars = 100 µm. [file MPP-20-1748-s006.jpg]

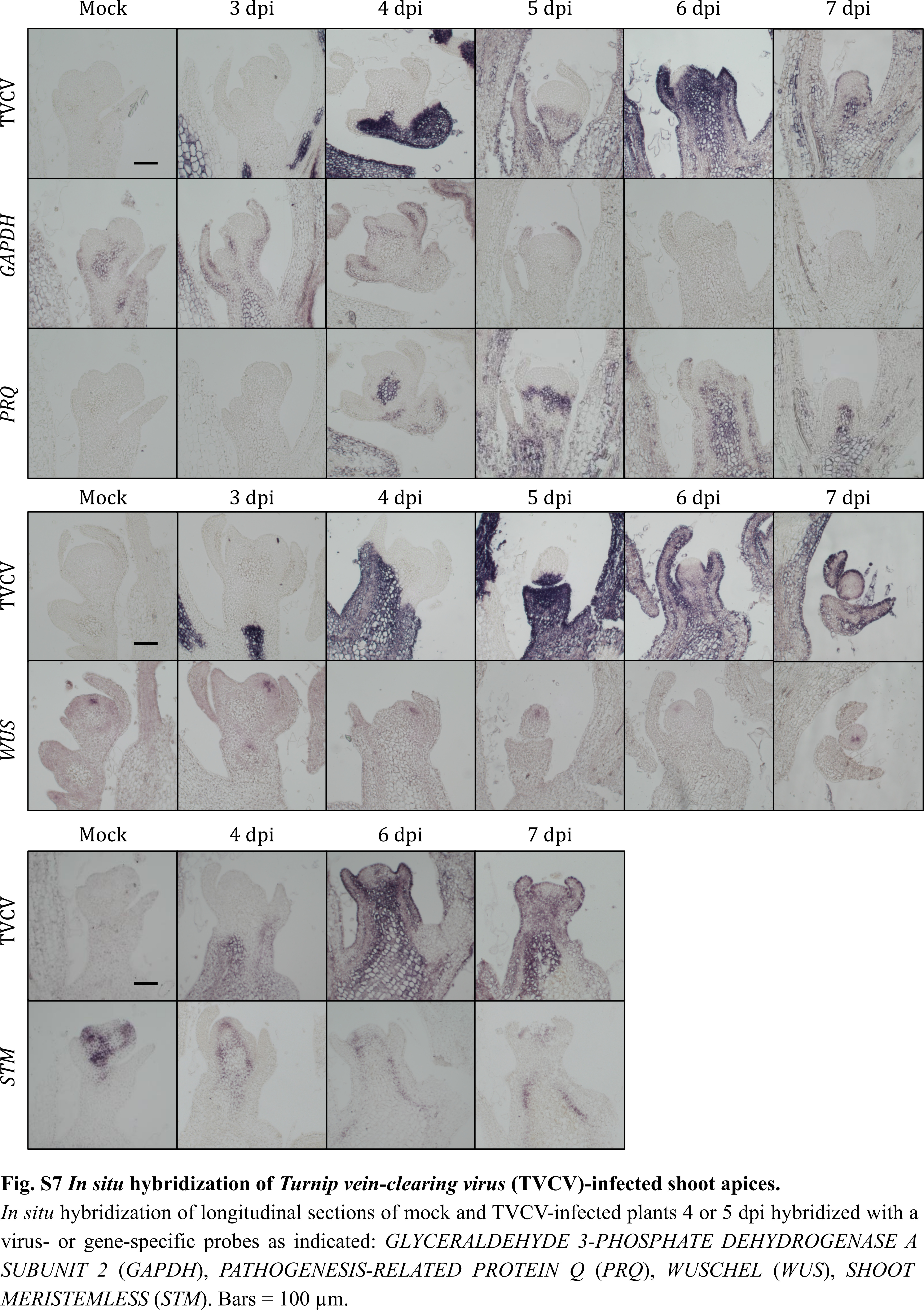

Supplement: Supplementary file 7 — Fig. S7 In situ hybridization of TVCV‐infected shoot apices. In situ hybridization of longitudinal sections of mock‐ and TVCV‐infected plants 4 or 5 dpi hybridized with a virus‐ or gene‐specific probes as indicated: GLYCERALDEHYDE 3‐PHOSPHATE DEHYDROGENASE A SUBUNIT 2 (GAPDH), PATHOGENESIS‐RELATED PROTEIN Q (PRQ), WUSCHEL (WUS), SHOOT MERISTEMLESS (STM). Bars = 100 µm. [file MPP-20-1748-s007.jpg]

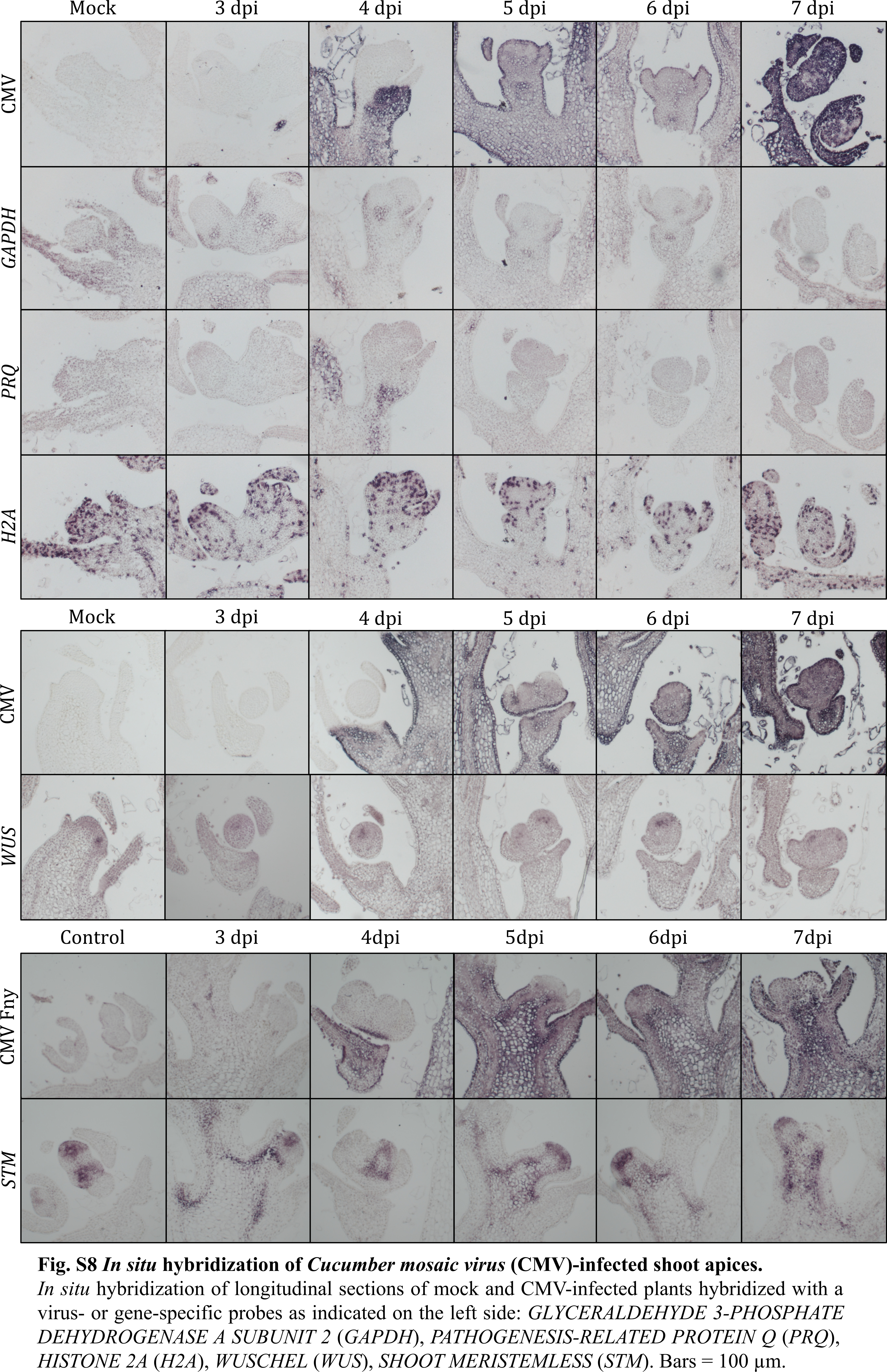

Supplement: Supplementary file 8 — Fig. S8 In situ hybridization of CMV‐infected shoot apices. In situ hybridization of longitudinal sections of mock‐ and CMV‐infected plants hybridized with a virus‐ or gene‐specific probes as indicated on the left side: GLYCERALDEHYDE 3‐PHOSPHATE DEHYDROGENASE A SUBUNIT 2 (GAPDH), PATHOGENESIS‐RELATED PROTEIN Q (PRQ), HISTONE 2A (H2A), WUSCHEL (WUS), SHOOT MERISTEMLESS (STM). Bars = 100 µm. [file MPP-20-1748-s008.jpg]

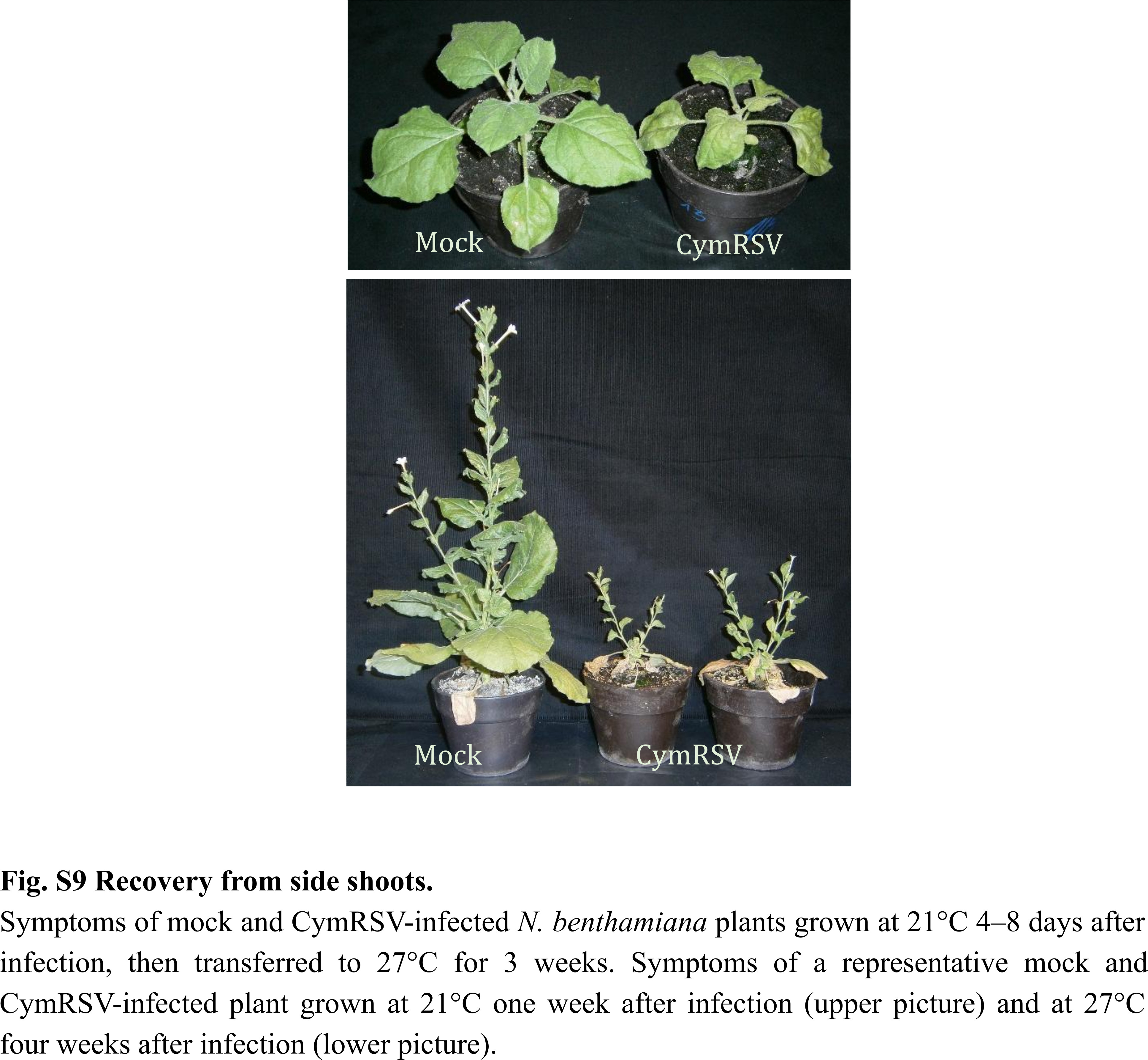

Supplement: Supplementary file 9 — Fig. S9 Recovery from side shoots. Symptoms of mock and CymRSV‐infected Nicotiana benthamiana plants grown at 21 °C 4–8 days after infection, then transferred to 27 °C for 3 weeks. Symptoms of a representative mock‐ and CymRSV‐infected plant grown at 21 °C one week after infection (upper picture) and at 27 °C four weeks after infection (lower picture). [file MPP-20-1748-s009.jpg]

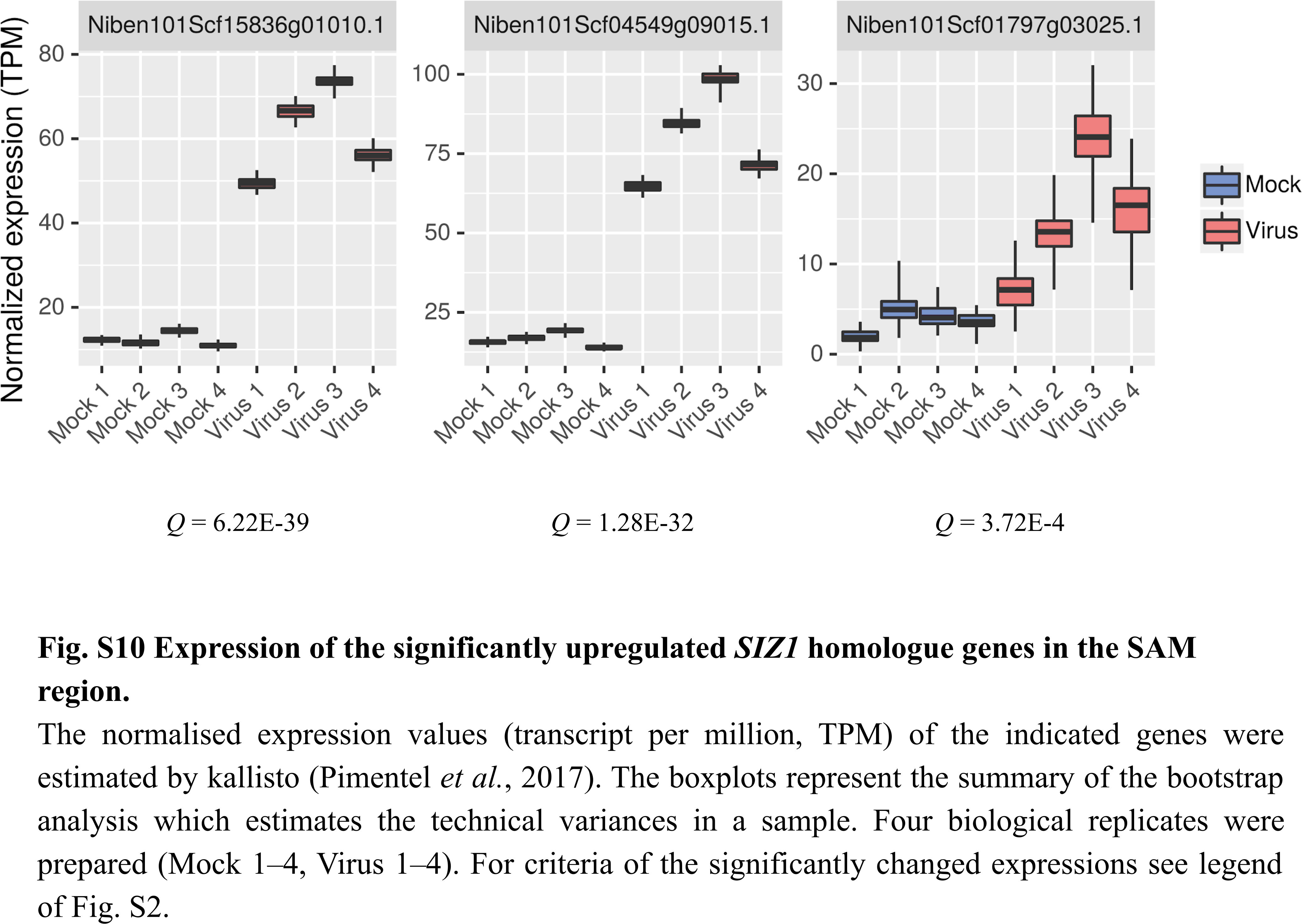

Supplement: Supplementary file 10 — Fig. S10 Expression of the significantly up‐regulated SIZ1 homologue genes in the SAM region. The normalized expression values (transcript per million, TPM) of the indicated genes were estimated by kallisto (Pimentel et al., 2017), log2‐transformed and z‐scores were calculated. The z‐scores show how many standard deviations the given value is above (red) or below (blue) from the mean (white) of all the values in the row. We show the values of the four biological replicates (Mock 1–4, Virus 1–4). For criteria of the significantly changed expressions see legend of Fig. S2. [file MPP-20-1748-s010.jpg]

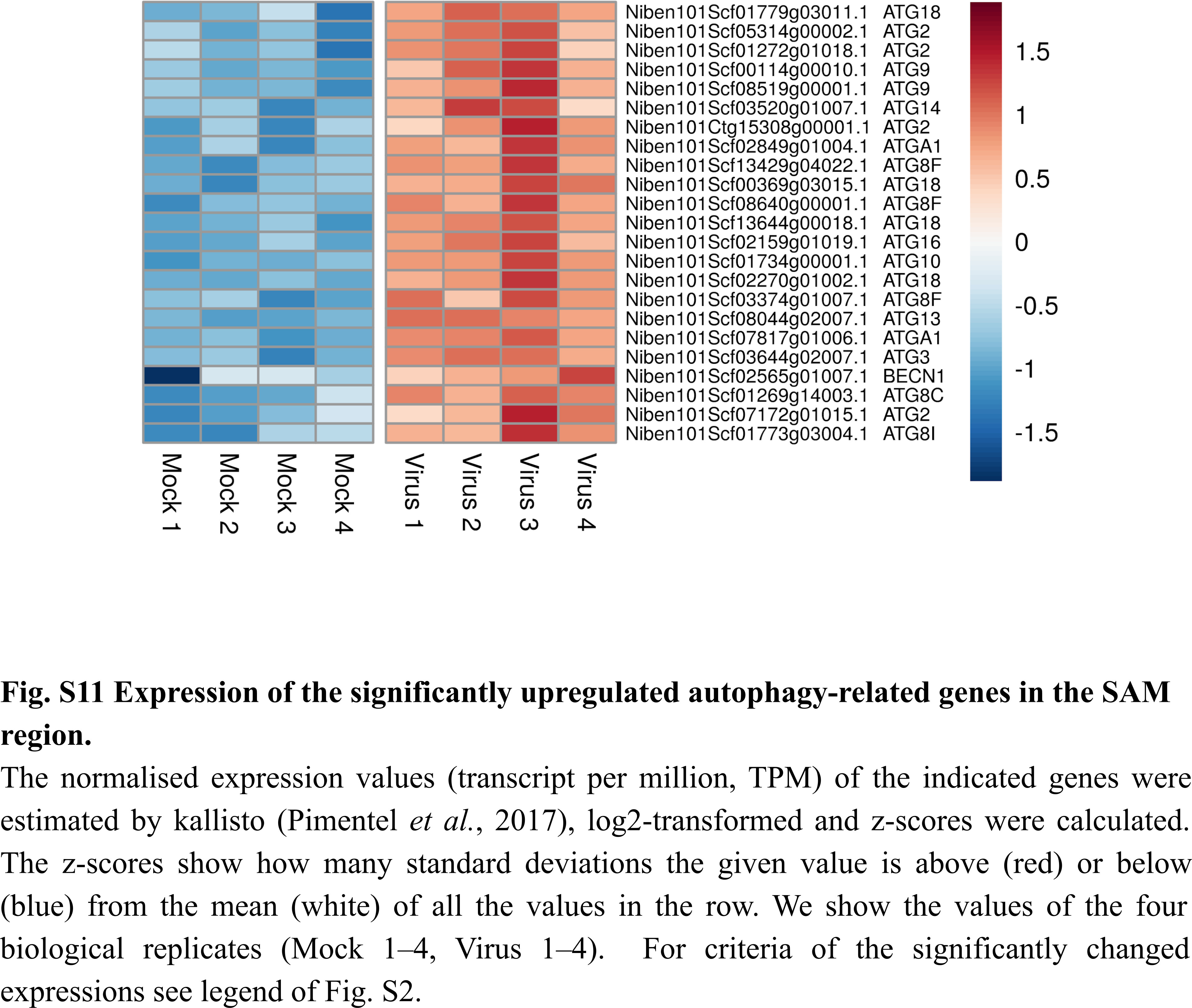

Supplement: Supplementary file 11 — Fig. S11 Expression of the significantly up‐regulated autophagy‐related genes in the SAM region. The normalized expression values (transcript per million, TPM) of the indicated genes were estimated by kallisto (Pimentel et al., 2017), log2‐transformed and z‐scores were calculated. The z‐scores show how many standard deviations the given value is above (red) or below (blue) from the mean (white) of all the values in the row. We show the values of the four biological replicates (Mock 1–4, Virus 1–4). For criteria of the significantly changed expressions see legend of Fig. S2. [file MPP-20-1748-s011.jpg]

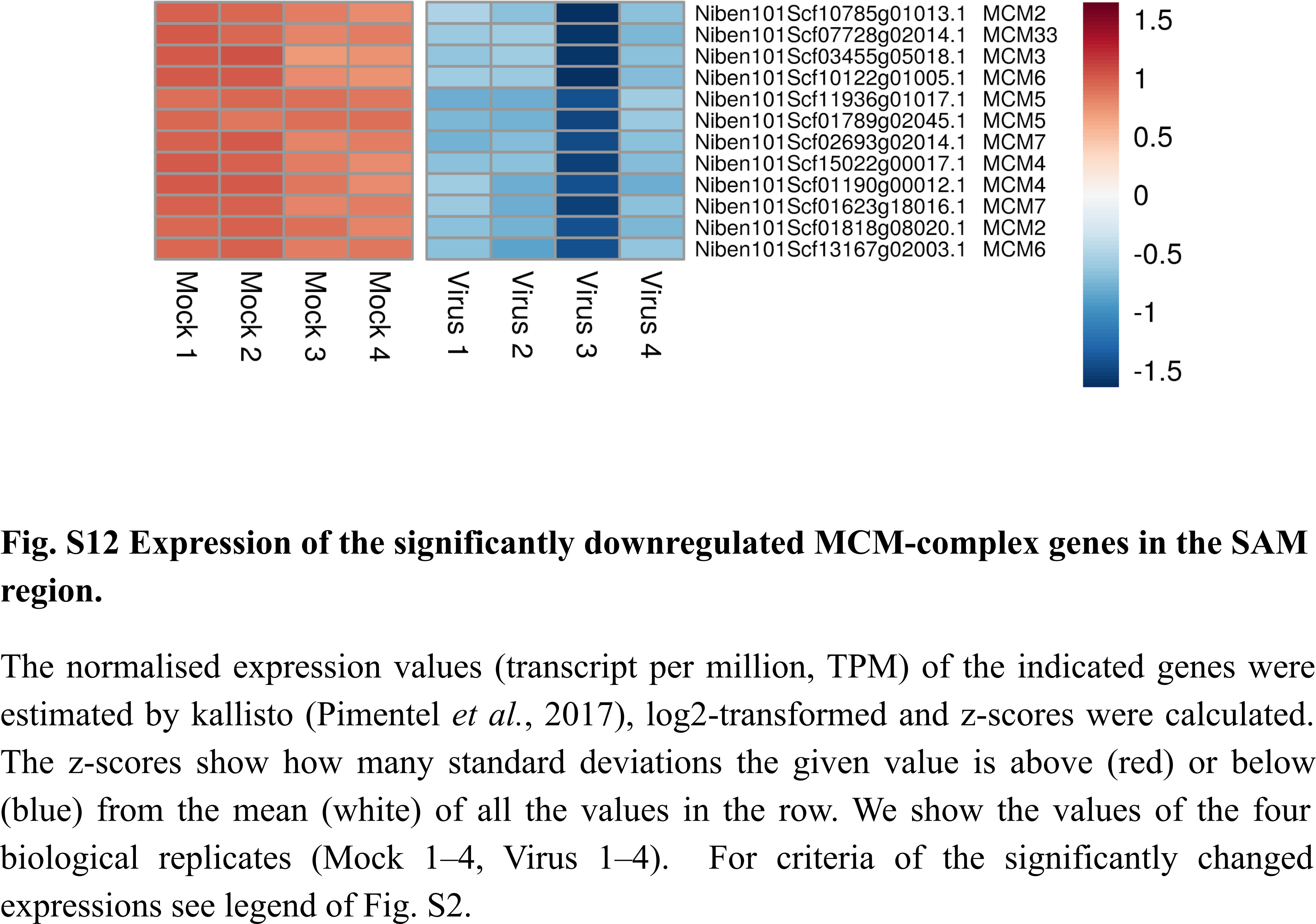

Supplement: Supplementary file 12 — Fig. S12 Expression of the significantly down‐regulated MCM‐complex genes in the SAM region. The normalized expression values (transcript per million, TPM) of the indicated genes were estimated by kallisto (Pimentel et al., 2017), log2‐transformed and z‐scores were calculated. The z‐scores show how many standard deviations the given value is above (red) or below (blue) from the mean (white) of all the values in the row. We show the values of the four biological replicates (Mock 1–4, Virus 1–4). For criteria of the significantly changed expressions see legend of Fig. S2. [file MPP-20-1748-s012.jpg]
